# miR-338-3p inhibits the invasion of renal cell carcinoma by downregulation of ALK5

**DOI:** 10.18632/oncotarget.19329

**Published:** 2017-07-18

**Authors:** Xiaoqian Zhang, Chunxia Wang, Hui Li, Xiaobin Niu, Xinwei Liu, Dongxu Pei, Xiaolan Guo, Xiaona Xu, Yongwei Li

**Affiliations:** ^1^ Department of Clinical Laboratory, Henan Province Hospital of TCM, The Second Affiliated Hospital of Henan University of Chinese Medicine, Zhengzhou 450002, Henan Province, China

**Keywords:** miR-338-3p, renal cell carcinoma (RCC), activin receptor-like kinase 5 (ALK5), invasion, metastasis

## Abstract

**Background:**

The current study aims to elucidate the role of miRNA-338-3p (miR-338-3p) in the invasion of renal cell carcinoma (RCC).

**Methods:**

Quantitative reverse transcription-polymerase chain reaction (qRT-PCR) was performed to detect the expression of miR-338-3p in human RCC cell lines with high metastatic potential (Caki-1) and low metastatic potential (786-O), respectively. The Caki-1 and 786-O cells were transfected with miR-338-3p mimic or inhibitor. Wound healing assay, Transwell assay and western blotting were performed to analyze the invasive ability and expression of activin receptor-like kinase 5 (ALK5) in the RCC cell lines. During the 36-month follow-up, we detected the expressions of miR-338-3p and ALK5 in 22 RCC cases with metastasis and 60 cases achieving a remission.

**Results:**

miR-339-3p was significantly downregulated in the Caki-1 cells as compared with the 786-O cells. The transfection with miR-338-3p inhibitor caused an increased invasive ability of both two cell lines. However, the transfection with miR-338-3p mimic caused a reduction of the invasiveness. In RCC cells, the expression of ALK5 was negatively correlated to miR-338-3p. Upregulation of ALK5 partially counteracted the miR-338-3p-induced invasiveness of RCC cells. We subsequently found the negative correlations between miR-338-3p and metastasis/ALK5 expression could be also observed in human RCC tissues.

**Conclusion:**

Taken together, these results indicate that miR-338-3p acts as a novel tumor suppressor to inhibit the invasion of RCC by regulating ALK5 expression.

## INTRODUCTION

Renal cell carcinoma (RCC), the predominant type of renal malignancy, is among the ten most common cancers [1, 2]. Although the availability of the treatments has been expanded in recent years, very limited tools can be used to predict local advanced and metastatic RCC [3–5]. Therefore, identifying the key molecules that control the invasion and metastasis of RCC is crucial for interventional therapies. microRNAs (miRNAs) can be divided into oncogenes and tumor suppressor genes based on their roles in cancers [6–8]. miRNA-338-3p (miR-338-3p) is involved in the invasion and metastasis of cancer cells [9]. To the best of our knowledge, the correlation between miR-338-3p and invasion and metastasis of RCC cells has not been clarified yet. To this end, we discussed the role of miR-338-3p in the invasion and metastasis of RCC. We have discovered miR-338-3p in RCC cells and clinical samples with varying metastatic potentials. Activin receptor-like kinase 5 (ALK5) can serve as the connector between the actin cytoskeleton and plasma membrane and participate in cell adhesion and cell motility [10]. Mounting evidences have demonstrated that ALK5 is involved in the metastasis of several human cancers. In this study, we hypothesized that miR-338-3p inhibits the invasion of RCC by regulating ALK5 and validated this hypothesis through experiments.

## RESULTS

### Expression of mi.R-338-3p in RCC cells with different metastatic potentials

**Figure 1:**
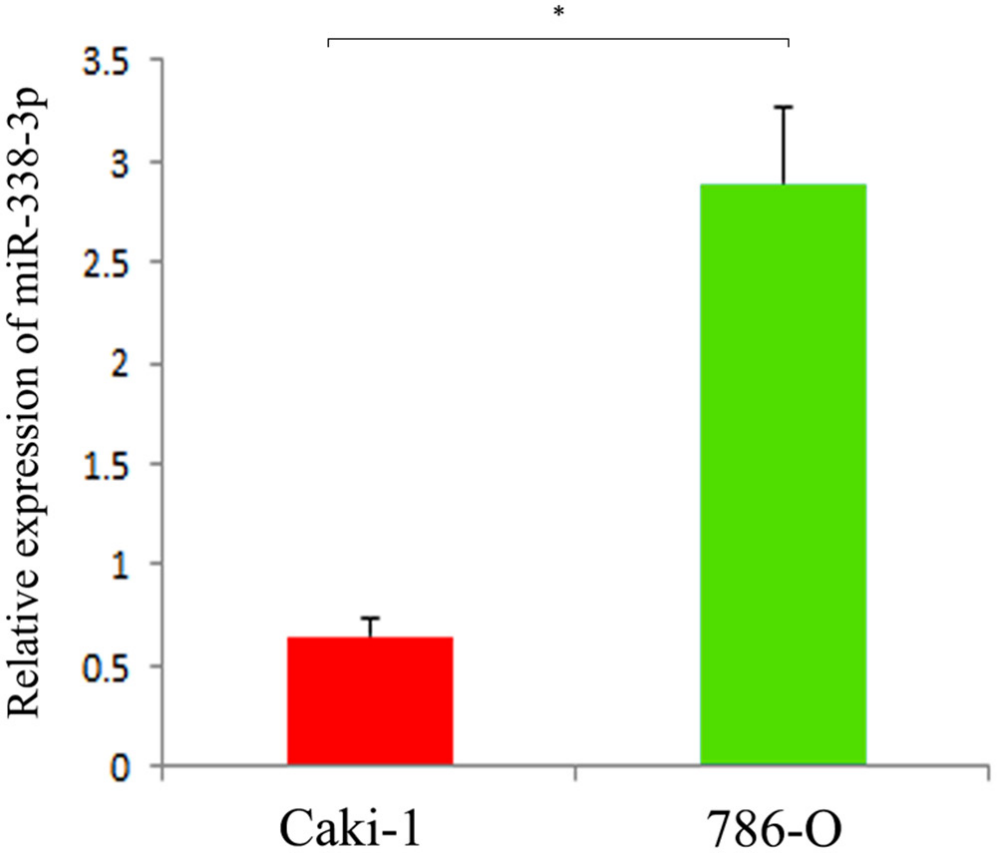
Expression of miR-338-3p in different metastatic potential RCC cell lines detected by qRT-PCR *p< 0.05 vs Caki-1 group.

We performed real-time quantitative PCR to determine the expressions of miR-338-3p in RCC cells with different metastatic potentials. The results showed that miR-338-3p was downregulated in Caki-1 cells with high metastatic potential and upregulated in 786-O cells with low metastatic potential (P<0.05, Figure 1). This finding indicated that the expression of miR-338-3p correlated to the metastatic phenotype of the RCC cells.

### Role of miR-338-3p in the migration of RCC cells *in vitro*

**Figure 2:**
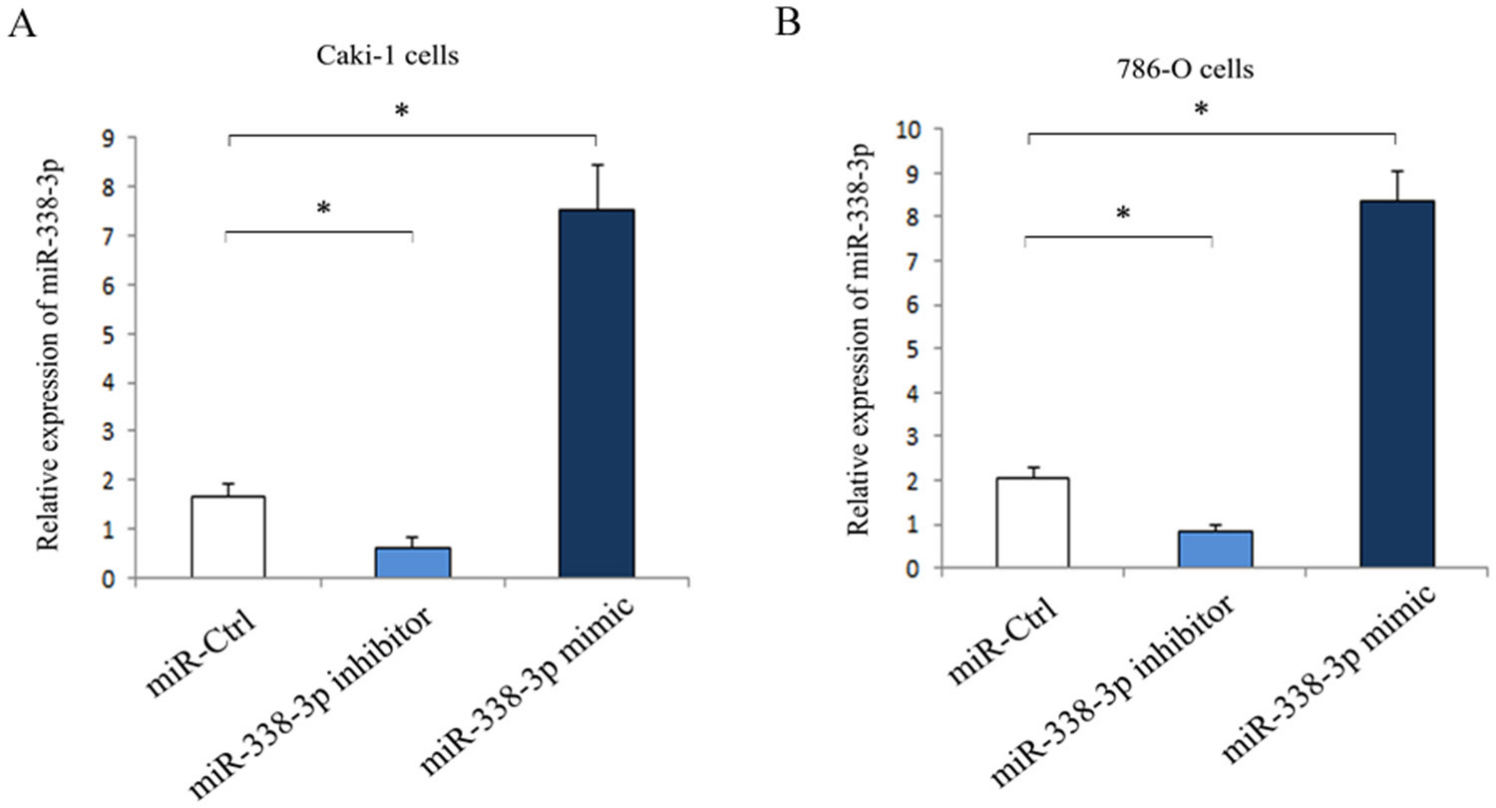
Expression of miR-338-3p in RCC cells post-transfection with miR-338-3p miR-338-3p mimic or inhibitor for 24 h **(A)** Caki-1 cells; **(B)** 786-O cells. *p< 0.05 vs miR-Ctrl group.

**Figure 3:**
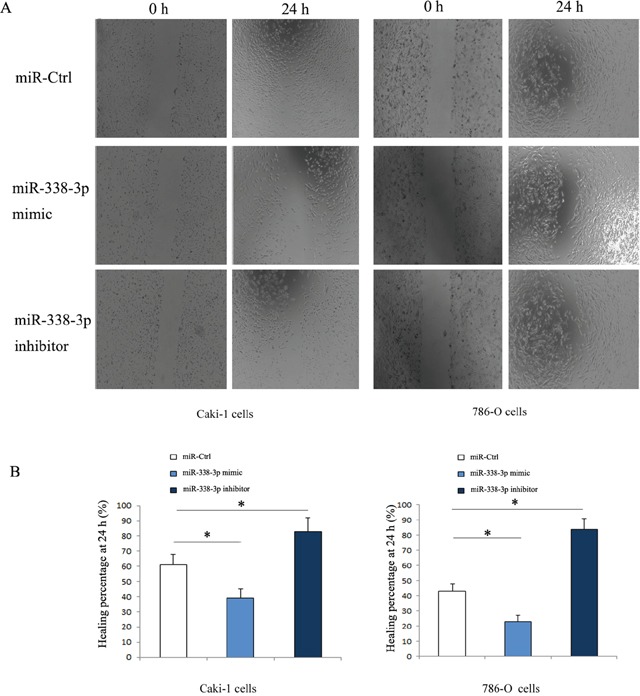
Migration of RCC cells transfected with miR-338-3p mimic or inhibitor detected by wound healing assay **(A)** The migrated cells increased in miR-338-3p inhibitor transfection group, while the migrated cells decreased in miR-338-3p mimic transfection group. **(B)** Healing percentage after transfection for 24 h was quantified. *p < 0.05 vs miR-Ctrl group.

As shown in Figure 2, the Caki-1 and 786-O cells transfected with miR-338-3p inhibitor were downregulated and the cells transfected with miR-338-3p mimic were upregulated significantly as compared with the cells transfected with miR-control. Wound healing assay was done to evaluate the effect of miR-338-3p expression on the migration of RCC cells *in vitro*. The results showed that as compared with the cells transfected with miR-negative control, Caki-1 and 786-O cells transfected with miR-338-3p mimic had a dramatic reduction in the migration capacity (Caki-1: 41.3% ± 2.5% vs. 64.4% ± 6.2%, P<0.05; 786-O: 27.6% ± 4.8% vs. 46.1 % ±5.7%, P<0.05; Figure 3). In contrast, the migration of cells transfected with miR-338-3p inhibitor was accelerated (Caki-1: 86.5 %±5.9 % vs. 62.7% ± 5.6%, P<0.05; 786-O; 88.0% ± 7.4% vs. 43.8% ± 7.2%, P<0.05; Figure 3).

### Effect of miR-338-3p on the invasion of RCC cells *in vitro*

**Figure 4:**
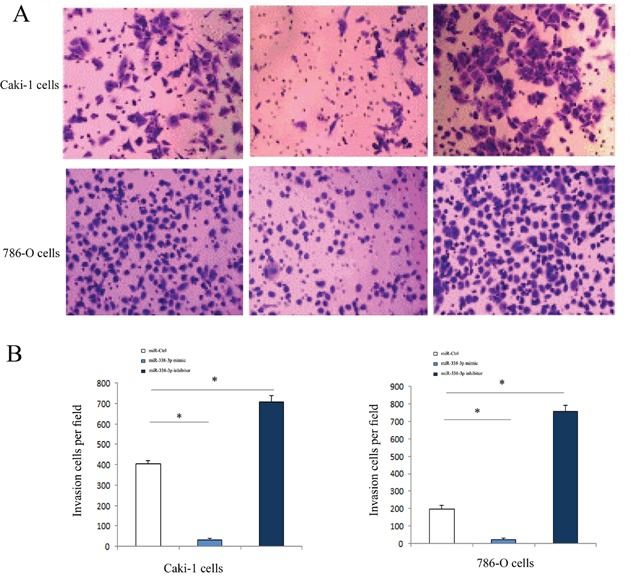
Invasive ability of of RCC cells transfected with miR-338-3p miR-338-3p mimic or inhibitor examined by Transwell assay **(A)** Invasive ability was increased in RCC cells in miR-338-3p inhibitor transfection group and was decreased in miR-338-3p mimic transfection group. **(B)** Quantitative analysis of invasive ability in each group. *p < 0.05 vs miR-Ctrl group.

Transwell assay results showed that, as compared with the cells transfected with miR-negative control the numbers of invaded Caki-1 and 786-O cells transfected with miR-338-3p inhibitor increased significantly (P<0.05). However, the numbers of cells invaded through the membrane after transfection with the miR-338-3p mimic decreased considerably (P<0.05, Figure 4).

### miR-338-3p inhibited the invasion of RCC cells by regulating the expression of ALK5

**Figure 5:**
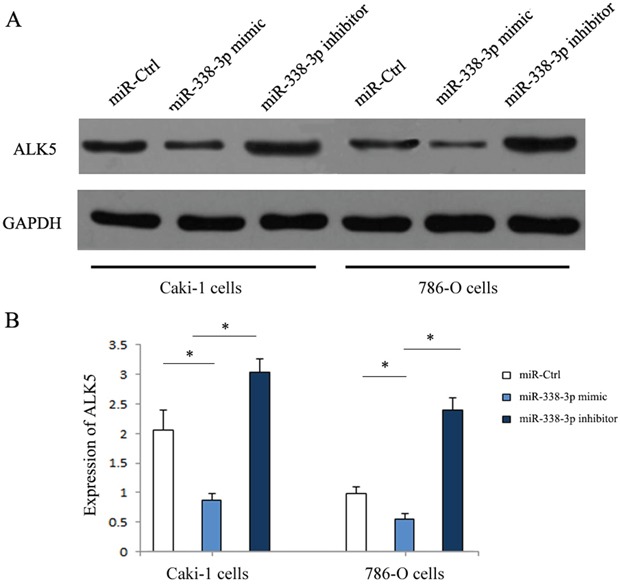
Expression of ALK5 in RCC cells transfected with miR-338-3p miR-338-3p mimic or inhibitor by western blotting **(A)** ALK5 was increased in RCC cells in miR-338-3p inhibitor transfection group and was decreased in miR-338-3p mimic transfection group. **(B)** Quantitative analysis of ALK5 expression in each transfection group. *p < 0.05 vs miR-Ctrl group.

**Figure 6:**
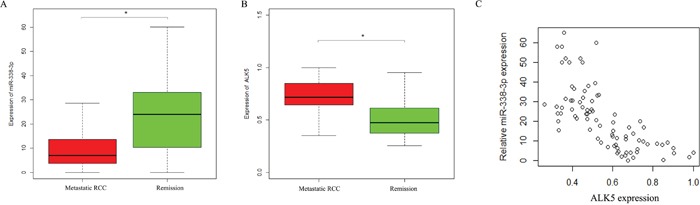
Expressions of ALK5 and miR-338-3p in metastatic and non-metastatic RCC patients detected by qRT-PCR **(A)** miR-338-3p was lower in patients with metastasis compared with patients with remission. * p < 0.05 vs miR-Ctrl group. **(B)** ALK5 was with metastasis compared with patients with remission. **(C)** The association between miR-338-3p and ALK5 in RCC patients by Pearson correlation analysis r = −0.67 (* p < 0.05).

Western Blot was performed to determine the expressions of ALK5 in the Caki-1and 786-O cells transfected with miR-338-3p mimic, miR-338-3p inhibitor or miR-control. ALK5 expressions were downregulated significantly in cells transfected with miR-338-3p mimic as compared with the cells transfected with miR-control (P<0.05). The expressions of ALK5 were upregulated significantly after transfection with miR-338-3p inhibitor (P<0.05, Figure 5). We further analyzed the correlation between miR-338-3p and ALK5 expressions and metastatic potential in a clinical environment by reviewing 82 patients with clear cell RCC. According to the 2010 AJCC TNM staging system, there were 48 patients with stage 1 RCC and 31 patients with stage 3 RCC. In the Fuhrman system, there were 11 patients of Furhman grade 1, 35 patients of Furhman grade 2, 24 patients of Furhman grade 3, and 12 patients of Furhman grade 4. During the follow-up that lasted for 36 months on average, 22 patients had metastasis, while the remaining 60 patients in remission (Figure 6). The results indicated that the metastatic patients had a downregulation of miR-338-3p (P<0.05) and an upregulation of ALK5 (P<0.05). Pearson's correlation coefficient that measured the statistical correlation ratio between the expressions of miR-338-3p and ALK5 was calculated as 0.69 (P<0.05).

## DISCUSSION

We found that the expression of miR-338-3p correlated negatively to the metastatic potential in RCC. An upregulation of miR-338-3p would cause a decrease in the invasiveness of the RCC cells. From these we infer that miR-338-3p may act as a tumor suppressor miRNA in RCC. To the best of our knowledge, this study was the first investigation into the correlation between miR-338-3p and invasiveness of RCC.

Some previous reports have focused on the role of miR-338-3p in other cancers [12]. Sun et al. showed that miR-338-3p directly targeted the human Ras-related protein Rab-14 (RAB14) oncogene and served as a tumor suppressive miRNA in human non-small cell lung carcinoma [13]. Specifically, miR-338-3p inhibited the epithelial-mesenchymal transition in gastric cancer by downregulating ZEB2 and MACC1/Met/Akt signaling [14]. Transfection with miR-338-3p caused a reduction in the proliferation, colony formation and migration of cancer cells expressing P-REX2a [15]. miR-338-3p was also an oncogenic miRNA in other tumors and promoted the invasion of tumor cells [16]. Xu et al. indicated that the expression of miR-338-3p in colorectal cancer was correlated positively to the metastasis of liver [17]. Therefore, miR-338-3p can serve as both tumor suppressor and oncogenic miRNA in different tumors. Some researchers have compared the miRNA expression profiles in metastatic and primary RCC by using the miRNA microarray technology [18–20]. miRNA expressions were compared between the metastatic and primary RCC in the same patient [21–23]. In another study, miRNA expressions were compared between the metastatic patients and the patients in remission during the follow-up, but no differential expression of miR-338-3p was indicated [24]. This inconsistency may be due to the limited sample size, which cannot completely remove the sampling bias.

Our results indicated that the expression of ALK5 correlated negatively to that of miR-338-3p. However, the specific influencing mechanism remains unknown. Besides, the regulatory role of miR-338-3p in the invasiveness of RCC may involve other pathways. Studies on the potentials of miR-338-3p in inhibiting the metastasis of RCC and in downregulating ALK5 will provide valuable clues for the prevention of the invasion and migration of RCC cells.

## CONCLUSION

In conclusion, the expression of miR-338-3p negatively correlates to the metastasis of RCC. Downregulation of miR-338-3p can inhibit the metastasis of RCC.

## MATERIALS AND METHODS

### Cell culture

Caki-1 and 786-O cells are human RCC cell lines with high and low metastatic potentials, respectively [11]. They were purchased from the American Type Culture Collection ATCC (Manassas VA, USA). The Caki-1 and 786-O cells were both maintained in RPMI-1640 medium (Gibco, Grand Island, New York, USA) containing 10% fetal bovine serum (FBS, Sangon Biotech (Shanghai) Co., Ltd.).

### Clinical samples

Human kidney samples were collected and assessed in an independent cohort of 82 RCC patients who received radical nephrectomy from January 2009 to September 2014. The collection and use of the kidney samples were approved by the ethics committee. Histological diagnosis was made according to the diagnostic criteria by WHO. The cases were selected based on the usability of the kidney samples, and they were not stratified by any known preoperative or pathological prognostic factors. All cases were followed up for an average of 36 months (range 8∼64 months). A total of 82 surgically resected kidney samples were collected from cases who had received no preoperative treatment under the guidance of one experienced pathologist. The samples were immediately preserved in liquid nitrogen prior to RNA or protein extraction. Written informed consent was obtained from all patients and the study was approved by the Ethics Committee of Henan Province Hospital of TCM, the Second Affiliated Hospital of Henan University of Chinese Medicine (Approval number: DYH2009026).

### Real-time quantitative fluorescence PCR

Total RNA extraction was performed using the TRIzol reagent according to the manufacturer's instruction. Hairpin-it™ miRNA qPCR Quantitation Kit was used to detect the expression of miR-338-3p. The sense primer sequence was 5′-TTAGTG TACCAGCCAT-3′ and the anti-sense primer was 5′-GAATGCGGGAGCGAA-3′. The synthetic miRNA standard included in the kit was taken as positive control; the U6 small nuclear RNA was the internal reference, and its sense primer was 5′-ACAAGTTGGATACAGAAGAGAT-3′ and anti-sense primer 5′-GGTACTCAGCAATAGCTT-3′ (Shanghai GenePharma Co., Ltd., Shanghai, China). Relative expressions of miR-338-3p in the cancer tissues and cell liens were calculated using the 2-öct method.

### Transfection

RCC cells were seeded into the 6-well plate and were respectively transfected with 5nM miR-338-3p inhibitor, miR-338-3p mimic or miR-control. The Caki-1 and 786-O cells were transfected with Lipofectamine 2000 (Invitrogen). miR-338-3p inhibitor (5′-GCAAAAAUUAGUGUGCGCCAAA-3′), miR-338-3p mimic (5′-UUUGAGCAGCACUCAUUUUUGC-3′) and miR-control (5′-CAGUAC UUUUAGUGUGUACAA-3′) were purchased from RiboBio (Guangzhou, China), the sequence of miR-negative control was heterogenous from any known human genome sequence so as to eliminate the potential non-sequence-specific effect.

### Wound healing assay

Caki-1 and 786-O cells were inoculated to grow into a single layer, respectively. The cells were respectively transfected with miR-338-3p mimic, miR-338-3p inhibitor or miR-control, and hungered by culture in a DMEM containing 0.1% FBS for 24h. A scratch was made in the culture flask using pipette. After that, the single layer of cells was washed twice to remove the non-adherent cells. The cells were further cultured in the medium containing 1% FBS for 24h, and the closure of the scratch was observed under the phase-inverted microscope. The average percentage of the cell-free area after cell culture to the initial denuded area was calculated using the Image J software.

### Invasion assay

After the transfection with miR-338-3p mimic, miR-338-3p inhibitor or miR-control, the invasiveness of the Caki-1 and 786-O cells was assessed by the Transwell assay. The Transwell chamber was covered with matrigel basement membrane. The lower and upper chambers were separated with polycarbonate membrane (Corning, NY, USA). The bottom of the chamber was filled with medium. The transfected cells were inoculated to the upper chamber and placed at 37°C in a 5% CO_2_ incubator. The cell coating was removed from the upper chamber with a cotton ball 40h later. The cells migrating to the reverse side of the membrane were stained with 0.05% crystal violet. Six fields of view were randomly selected by two independent pathologists. The cells were counted in each field of view and the average was taken. The experiments were performed in triplicate. In order to analyze the inhibitory role of ALK5 on RCC under the regulation by miR-338-3p, the Caki-1 and 786-O cells were pretreated with 10 μM IN-1130, a small-molecule ALK5 inhibitor, for 30 min, respectively. Then the cells were transfected with miR-control or miR-338-3p inhibitor for 24h.

### Western blot

The proteins were separated by sodium dodecyl sulfate (SDS)-8% polyacrylamide gel electrophoresis, and blotted to the PVDF membrane (Bio-Rad, Hercules, California, USA). Immunoassay was performed using ALK5 antibody probe. The membrane was incubated with peroxidase-coupled secondary antibody (Abcam, Cambridge, MA, U.S.). After washing by PBS for 15 min, protein bands were detected using the ECL Western Blotting Kit (Pierce Chemical, Rockford, IL, USA). The relative protein expression was analyzed by Image-Pro plus software 6.0, represented as the density ratio versus GAPDH.

### Statistical analysis

All data were analyzed using SPSS 16.0 software (SPSS, Chicago, IL, United States). Data were expressed as mean±standard deviation. Both the negative and positive controls were used for quality control in the conventional manner. One way ANOVA were performed for comparing the differences among the groups. P<0.05 indicated significant differences.

## Abbreviations

RCC: renal cell carcinoma
qRT-PCR: quantitative reverse transcription-polymerase chain reaction
ALK5: activin receptor-like kinase 5
miRNAs: microRNAs
